# Role of the COVID-19 Pandemic on Sexual Behaviors and Receipt of Sexual and Reproductive Health Services Among U.S. High School Students — Youth Risk Behavior Survey, United States, 2019–2021

**DOI:** 10.15585/mmwr.su7201a7

**Published:** 2023-04-28

**Authors:** Leigh E. Szucs, Sanjana Pampati, Jingjing Li, Casey E. Copen, Emily Young, Sandra Leonard, DNP, Michelle N. Carman-McClanahan

**Affiliations:** ^1^Division of Adolescent and School Health, National Center for HIV, Viral Hepatitis, STD, and TB Prevention, CDC; ^2^Division of STD Prevention, National Center for HIV, Viral Hepatitis, STD, and TB Prevention, CDC; ^3^Oak Ridge Institute for Science and Education, Oak Ridge, Tennessee

## Abstract

Disproportionate rates of sexually transmitted diseases (STDs), including HIV, and unintended pregnancy among adolescents persist and might have been affected by the COVID-19 pandemic. This study uses 2019 and 2021 data from the nationally representative Youth Risk Behavior Surveys to characterize changes in sexual behaviors and receipt of sexual and reproductive health services among U.S. high school students before and during the pandemic. Outcomes included HIV testing (lifetime), STD testing (past 12 months), condom use (last sexual intercourse), and primary contraceptive method used to prevent pregnancy (last sexual intercourse). Except for HIV testing, all analyses were limited to currently sexually active students. Weighted prevalence and 95% CIs of outcomes for 2019 and 2021 were calculated for each year by demographics (sex [female or male], age, and race and ethnicity) and sex of sexual contacts (opposite sex only, both sexes, same sex only). For each year, pairwise *t*-tests with Taylor series linearization were used to identify demographic differences among outcomes. Across years, change in prevalence of outcomes was assessed by using absolute and relative measures of association overall and by demographics. During 2019–2021, the prevalence of HIV testing decreased by 3.68 percentage points, from 9.4% to 5.8%. Among sexually active students, prevalence of STD testing decreased by 5.07 percentage points, from 20.4% to 15.3%. Among sexually active students reporting opposite sex or both sexes sexual contact, intrauterine device or implant use at last sexual intercourse increased by 4.11 percentage points, from 4.8% to 8.9%, and no contraceptive method use increased by 2.74 percentage points, from 10.7% to 13.4%. Because of disruptions throughout the pandemic, results underscore the importance of improving access to a range of health services for adolescents and improving STD/HIV and unintended pregnancy prevention.

## Introduction

Disproportionate rates of sexually transmitted diseases (STDs), including HIV and unintended pregnancy, have long affected adolescents and young adults across the United States. High rates of annual chlamydia cases among females aged 15–19 years (https://www.cdc.gov/std/statistics/2021/default.htm), new HIV infections among adolescent sexual minority males (https://www.cdc.gov/hiv/library/reports/hiv-surveillance.html), and higher rates of adolescent pregnancy and birth in the United States compared with other high-income countries are of concern (*1*). Despite declines in pregnancy and birth rates and improvements in contraceptive use among adolescents (*2*), racial and ethnic, geographic, and socioeconomic disparities persist (*3*). Studies highlight increases in condomless sex among Black or African American (Black) and White youths (*3*), decreases in contraceptive method use during sexual intercourse (*2*,*4*), and suboptimal adherence to STD testing among adolescent females (*5*). Such data on the sexual and reproductive health (SRH) behaviors and experiences were collected before the 2020 onset of the COVID-19 pandemic and do not reflect the stressors and disruptions experienced by adolescents during the pandemic.

Beginning in early 2020, nationwide stay-at-home orders, physical distancing, quarantine and isolation guidance, and disruptions in access to SRH services had substantial effects on adolescent and adult sexual health (*6*). During the pandemic, decreases or total elimination of in-person health care visits, including facility closures, discontinuation of prevention and treatment methods (e.g., pre-exposure prophylaxis and birth control), and physical and economic barriers to preventative services (e.g., transportation and insurance coverage) were reported by older adolescents and adults (*7*). Income or health insurance coverage loss by adolescents or their parents might have created economic barriers to accessing and paying for health services (e.g., contraceptives) (*6*). To date, limited studies have explicitly examined adolescents’ SRH behaviors and experiences before and during the pandemic (*6*,*8*). To address this gap, this study uses 2019 and 2021 Youth Risk Behavior Survey (YRBS) data to describe prevalence estimates of sexual behaviors and receipt of SRH services among U.S. high school students during the pandemic. Because of disruptions in access to health services caused by the pandemic, this study primarily focused on adolescents’ reported receipt of SRH services that were primarily accessible through health care providers (i.e., testing and hormonal contraception) and sexual behaviors, examining differences by demographic characteristics and absolute and relative changes over time. Results can support public health goals for reducing or preventing STD/HIV and unintended pregnancy and addressing disparities in access to and quality of adolescent SRH preventative care.

## Methods

### Data Source

This report includes data from the 2019 (N = 13,677) and 2021 (N = 17,232) YRBS, a cross-sectional, school-based survey conducted biennially since 1991. Each survey year, CDC collects data from a nationally representative sample of public and private school students in grades 9–12 in the 50 U.S. states and the District of Columbia. Additional information about YRBS sampling, data collection, response rates, and processing is available in the overview report of this supplement (*9*). The prevalence estimates for sexual behaviors and receipt of SRH services for the overall study population and by sex, race and ethnicity, grade, and sexual identity are available at https://nccd.cdc.gov/youthonline/App/Default.aspx. The full YRBS questionnaire, data sets, and documentation are available at https://www.cdc.gov/healthyyouth/data/yrbs/index.htm. This activity was reviewed by CDC and was conducted consistent with applicable federal law and CDC policy.*

### Measures

Students’ self-reported sexual behaviors and receipt of SRH services, were examined as outcomes (Table 1). Lifetime HIV testing and STD testing during the past 12 months were both dichotomized as yes or no/don’t know. Any condom use at last sexual intercourse was dichotomized as yes or no. Four measures were constructed to capture use and nonuse of primary contraceptive method to prevent pregnancy at last sexual intercourse: 1) use of intrauterine device (IUD) or implant; 2) use of shot, patch, or birth control ring; 3) use of birth control pills; and 4) no contraceptive method used. These contraceptive methods were examined because of the study’s objective to explore changes in adolescents’ receipt of SRH services commonly accessible through a health care provider before and during the pandemic. Other primary contraceptive methods used to prevent pregnancy are also measured in YRBS but are not examined in this study (e.g., condoms as primary contraceptive method used to prevent pregnancy; 2021 = 45.3%).

**TABLE 1:** Measures for select sexual and reproductive health services and sexual behaviors among high school students — Youth Risk Behavior Survey, United States, 2019–2021*

Construct	Measure	Response option
**Receipt of SRH services**		
HIV testing, lifetime	Have you ever been tested for HIV, the virus that causes AIDS? (Do not count tests done if you donated blood.)	Yes, no, not sure
STD testing, past 12 months	During the past 12 months, have you been tested for an STD other than HIV, such as chlamydia or gonorrhea?	Yes, no, not sure
**Sexual behaviors**		
Condom use at last sexual intercourse	The last time you had sexual intercourse, did you or your partner use a condom?	I have never had sexual intercourse, yes, no
Primary contraceptive method to prevent pregnancy:^†^ Use of IUD or implant at last sexual intercourse Use of shot, patch, or birth control ring at last sexual intercourse Use of birth control pills at last sexual intercourse Use of no contraceptive method at last sexual intercourse	**2019**: The last time you had sexual intercourse, what one method did you or your partner use to prevent pregnancy? (Select only one response.) **2021**: The last time you had sexual intercourse with an opposite-sex partner, what one method did you or your partner use to prevent pregnancy? (Select only one response.)	An IUD (such as Mirena or ParaGard) or implant (such as Implanon or Nexplanon) before last sexual intercourse with an opposite-sex partner (to prevent pregnancy among students who were currently sexually active); shot (such as Depo-Provera), patch (such as Ortho Evra), or birth control ring (such as NuvaRing); birth control pills (**2019**), birth control pills (Do not count emergency contraception such as Plan B or the “morning after” pill.) (**2021**); no method to prevent pregnancy

**Abbreviations**: IUD = intrauterine device; SRH = sexual and reproductive health; STD = sexually transmitted disease.

* 2019: N = 13,677 respondents; 2021: N =17,232 respondents. Because the state and local questionnaires differ by jurisdiction, students in these schools were not asked all national YRBS questions. Therefore, the total number (N) of students answering each question varied. Percentages in each category are calculated on the known data.

^†^ YRBS question wording and response options for primary contraceptive method used to prevent pregnancy at last sexual intercourse changed between the 2019 and 2021 surveys. Question wording and response options are reported for both years when there were differences. Full survey instruments can be found at https://www.cdc.gov/healthyyouth/data/yrbs/questionnaires.htm.

Demographic characteristics examined included sex (female or male), age (aged ≤15 years, 16–17 years, or ≥18 years), and race and ethnicity. Race and ethnicity was categorized as Black, Hispanic or Latino (Hispanic), and White. (Persons of Hispanic origin might be of any race but are categorized as Hispanic; all racial groups are non-Hispanic.) The number of students from other race or multiracial groups was too small for separate analyses. Sex of lifetime sexual contacts was categorized as opposite sex only, same sex only, or both sexes.

### Analysis

For lifetime HIV testing, the analytic sample was not restricted. For STD testing during the past 12 months and any condom use at last sexual intercourse, the analytic sample was restricted to those who are currently sexually active (i.e., those who reported having had sexual intercourse with one or more persons during the 3 months before survey administration). The overall YRBS sample included 27.0% and 21.6% of students reporting being currently sexually active in 2019 and 2021, respectively. For analyses examining primary contraceptive method use to prevent pregnancy at last sexual intercourse, the analytic sample was restricted to currently sexually active students reporting opposite sex or both sexes sexual contacts during their lifetime.

Weighted prevalence and 95% CIs of sexual behaviors and receipt of SRH services are presented for each year (2019 and 2021) and by demographic characteristics. Within the same year, demographic differences in prevalence of sexual behaviors and receipt of SRH services were examined by using pairwise *t*-tests with Taylor series linearization. Across years, changes in sexual behaviors and receipt of SRH services from 2019 to 2021 were assessed by using absolute (prevalence difference [PD]) and relative (prevalence ratio [PR]) measures among the total sample, as well as by demographic characteristics. Models used to obtain PD and PR estimates did not control for any demographic variables or other covariates. P-values <0.05 and 95% CIs that did not cross zero (for PD) or 1.0 (for PR) were considered statistically significant. Prevalence estimates with a denominator <30 were considered statistically unreliable and therefore were suppressed (*9*). Analyses were conducted using SAS-callable SUDAAN (version 11.0.3; RTI International) to account for the complex sampling design, and weights were applied to account for school and student nonresponse and to represent the U.S. high school student population.

## Results

From 2019 to 2021, the prevalence of lifetime HIV testing decreased significantly by 3.68 percentage points, from 9.4% to 5.8% (Table 2). Among currently sexually active students, the prevalence of STD testing during the past 12 months decreased significantly by 5.07 percentage points, from 20.4% to 15.3%; condom use at last sexual intercourse did not significantly change. Among currently sexually active students reporting opposite sex or both sexes sexual contacts during their lifetime, the prevalence of IUD or implant use at last sexual intercourse increased significantly by 4.11 percentage points, from 4.8% to 8.9%, and no contraceptive method use increased significantly by 2.74 percentage points, from 10.7% to 13.4%. Among currently sexually active students reporting opposite sex or both sexes’ sexual contacts during their lifetime, shot, patch, or birth control ring use and use of birth control pills (all at last sexual intercourse) did not significantly change over time.

**TABLE 2:** Changes in prevalence of sexual and reproductive health services and sexual behaviors among high school students, overall and by sex — Youth Risk Behavior Survey, United States, 2019–2021

Sexual and reproductive health service and sexual behavior (n)*	Total				Female				Male			
	2019 % (95% CI)	2021 % (95% CI)	PD % (95% CI)^†^	PR % (95% CI)^†^	2019 % (95% CI)	2021 % (95% CI)	PD % (95% CI)^†^	PR % (95% CI)^†^	2019 % (95% CI)	2021 % (95% CI)	PD % (95% CI)^†^	PR % (95% CI)^†^
**HIV testing, lifetime (n = 25,033)**	9.4 (8.5 to 10.4)	5.8 (5.1 to 6.5)	–3.68 (–4.84 to –2.52)^†^	0.61 (0.52 to 0.71)^†^	10.0 (9.0 to 11.1)	5.8 (5.0 to 6.7)	–4.24 (–5.63 to –2.85)^§^	0.58 (0.48 to 0.69)^¶^	8.8 (7.7 to 10.0)**	5.7 (5.0 to 6.5)	–3.10 (–4.48 to –1.71)^††^	0.65 (0.54 to 0.78)^§§^
**STD testing, past 12 months (n = 4,559)**	20.4 (17.5 to 23.6)	15.3 (13.4 to 17.5)	–5.07 (–8.71 to –1.44)^†^	0.75 (0.62 to 0.92)^†^	26.1 (22.5 to 30.2)	17.6 (14.6 to 20.9)	–8.58 (–13.56 to –3.59)^§^	0.67 (0.53 to 0.85)^¶^	13.7 (11.1 to 16.9)**	12.7 (10.3 to 15.6)^¶¶^	1.04 (–4.97 to 2.88)	0.92 (0.69 to 1.24)
Condom use at last sexual intercourse (n = 6,455)***	54.3 (52.0 to 56.6)	51.8 (49.4 to 54.3)	–2.45 (–5.81 to 0.90)	0.95 (0. 90 to 1.02)	49.6 (45.6 to 53.6)	47.3 (43.2 to 51.4)	–2.30 (–8.01 to 3.40)	0.95 (0.85 to 1.07)	60.0 (57.0 to 62.9)**	57.7 (52.8 to 62.5)^¶¶^	–2.31 (–7.99 to 3.38)	0.96 (0.87 to 1.06)
**Use of IUD or implant to prevent pregnancy at last sexual intercourse (n = 5,171)**	4.8 (3.3 to 6.9)	8.9 (6.1 to 12.8)	4.11 (0.39 to 7.94)^†^	1.86 (1.10 to 3.12)^†^	5.6 (4.0 to 7.6)	10.3 (6.9 to 15.1)	4.76 (0.35 to 9.17)^§^	1.86 (1.12 to 3.07)^¶^	4.0 (2.1 to 7.4)	7.3 (4.5 to 11.5)	3.27 (–0.97 to 7.51)	1.82 (0.83 to 3.98)
**Use of shot, patch, or birth control ring to prevent pregnancy at last sexual intercourse (n = 4,796)**	3.5 (2.5 to 4.9)	3.5 (2.7 to 4.6)	0.04 (–1.46 to 1.54)	1.01 (0.66 to 1.55)	4.7 (3.1 to 7.1)	4.6 (3.4 to 6.2)	–0.12 (–2.51 to 2.26)	0.97 (0.59 to 1.62)	2.2 (1.3 to 3.7)**	2.3 (1.3 to 4.0)^¶¶^	0.13 (–1.59 to 1.85)	1.06 (0.49 to 2.28)
**Use of birth control pills to prevent pregnancy at last sexual intercourse (n = 5,171)**	23.3 (19.9 to 27.1)	20.8 (18.6 to 23.2)	–2.52 (–6.82 to 1.79)	0.89 (0.74 to 1.08)	26.1 (22.2 to 30.4)	22.8 (19.2 to 26.8)	–3.29 (–8.92 to 2.33)	0.87 (0.69 to 1.10)	20.2 (16.5 to 24.6)**	18.4 (15.7 to 21.4)	–1.87 (–6.85 to 3.11)	0.91 (0.70 to 1.17)
**Use of no contraceptive method to prevent pregnancy at last sexual intercourse (n = 5,171)**	10.7 (8.8 to 12.8)	13.4 (11.8 to 15.2)	2.74 (0.15 to 5.34)^†^	1.26 (1.01 to 1.57)^†^	11.9 (9.2 to 15.2)	15.2 (12.8 to 18.0)	3.33 (0.63 to 7.30)	1.28 (0.94 to 1.74)	9.3 (7.2 to 12.1)	11.3 (9.6 to 13.1)^¶¶^	1.92 (–1.09 to 4.93)	1.21 (0.89 to 1.64)

**Abbreviations:** IUD = intrauterine device; PD= prevalence difference; PR = prevalence ratio; STD = sexually transmitted disease.

* 2019: N = 13,677 respondents; 2021: N = 17,232 respondents. Because the state and local questionnaires differ by jurisdiction, students in these schools were not asked all national YRBS questions. Therefore, the total number (N) of students answering each question varied. Percentages in each category are calculated on the known data. Unweighted counts indicating denominators. For HIV testing (lifetime), all students were included in the sample. For any condom use at last sexual intercourse and STD testing (past 12 months), sample was restricted to currently sexually active (i.e., having had sexual intercourse with at least one person during the 3 months before the survey). For use of IUD or implant, shot, patch, or birth control ring, birth control pills, and no contraceptive method, sample was restricted to currently sexually active and those reporting opposite sex or both sex sexual contacts (lifetime).

^†^ PD and PR compare 2019 versus 2021. 95% CIs that did not cross zero (for PD) or 1.0 (for PR) were considered statistically significant (p<0.05).

^§^ PD comparing 2019 versus 2021 among females indicated a significant difference (p<0.05).

^¶^ PR comparing 2019 versus 2021 among females indicated a significant difference (p<0.05).

** Male students significantly differed from female students in 2019, based on *t*-test with Taylor series linearization (p<0.05).

^††^ PD comparing 2019 versus 2021 among males indicated a significant difference (p<0.05).

^§§^ PR comparing 2019 versus 2021 among males indicated a significant difference (p<0.05).

^¶¶^ Male students significantly differed from female students in 2021, based on *t*-test with Taylor series linearization (p<0.05).

*** Condom use at last sexual intercourse was measured by a separate item from the item for primary contraceptive method used for preventing pregnancy.

Among female students, STD testing and HIV testing significantly decreased by 8.58 percentage points and 4.24 percentage points, respectively: IUD or implant use significantly increased by 4.76 percentage points (Table 2). Among male students, HIV testing significantly decreased by 3.10 percentage points. In 2021, male students were less likely to report STD testing than female students (12.7% versus 17.6%). In 2021, male students were more likely to report condom use than female students (57.7% versus 47.3%). In 2021, compared with female students’ report of contraceptive method used (self or partner), male students were less likely to report they or their partner used no contraceptive method (15.2% versus 11.3%) and shot, patch, or birth control ring (4.6% versus 2.3%) at last sexual intercourse.

Lifetime HIV testing significantly decreased by 2.96 and 3.05 percentage points for students aged ≤15 and 16–17 years, respectively, but did not significantly change for students aged ≥18 years (Table 3). STD testing during the past 12 months decreased significantly by 12.22 percentage points, from 25.4% to 13.2% for students aged ≥18 years but did not significantly change for students aged ≤15 or 16–17 years. Among students aged 16–17 years, IUD or implant use at last sexual intercourse increased significantly by 6.29 percentage points, from 4.7% to 11.0% but did not significantly change for students aged ≤15 or ≥18 years. Among high school students aged ≤15 years, no contraceptive method used at last sexual intercourse increased significantly by 8.01 percentage points, from 12.4% to 20.5%, but did not significantly change for students aged 16–17 or ≥18 years.

**TABLE 3:** Changes in prevalence of sexual and reproductive health services and sexual behaviors and among high school students, by age — Youth Risk Behavior Survey, United States, 2019–2021

Sexual and reproductive health services and sexual behavior (n)*	≤15 years				16–17 years				≥18 years			
	2019 % (95% CI)	2021 % (95% CI)	PD % (95% CI)^†^	PR % (95% CI)^†^	2019 % (95% CI)	2021 % (95% CI)	PD % (95% CI)^†^	PR % (95% CI)^†^	2019 % (95% CI)	2021 % (95% CI)	PD % (95% CI)^†^	PR % (95% CI)^†^
**HIV testing, lifetime (n = 25,033)**	6.5 (5.2 to 8.1)	3.5 (2.7 to 4.5)	–2.96 (–4.68 to –1.24)^§^	0.54 (0.39 to 0.76)^¶^	10.3 (9.2 to 11.4)**	7.2 (6.2 to 8.4)^††^	–3.05 (–4.58 to –1.52)^§§^	0.70 (0.59 to 0.84)^¶¶^	13.8 (11.9 to 16.1)**^,†††^	10.3 (7.6 to 13.7)^§§§^	–3.58 (–7.25 to 0.09)	0.74 (0.53 to 1.08)
**STD testing, past 12 months (n = 4,559)**	13.0 (9.2 to 17.9)	8.4 (5.3 to 13.1)	–4.53 (–10.27 to 1.20)	0.65 (0.37 to 1.14)	20.6 (16.9 to 24.8)**	18.2 (16.0 to 20.6)^††^	–2.41 (–6.99 to 2.18)	0.88 (0.70 to 1.11)	25.4 (21.8 to 29.4)***	13.2 (8.4 to 20.1)	–12.22 (–19.12 to –5.31)^¶¶¶^	0.52 (0.33 to 0.82)^****^
**Condom use at last sexual intercourse (n = 6,455)^††††^**	59.7 (54.6 to 64.6)	55.1 (49.9 to 60.1)	–4.65 (–11.82 to 2.53)	0.92 (0.81 to 1.05)	54.2 (51.0 to 57.5)**	51.8 (48.3 to 55.3)	–2.42 (–7.23 to 2.39)	0.96 (0.87 to 1.05)	50.7 (45.8 to 55.6)***	46.4 (39.7 to 53.2)^§§§^	–4.31 (–12.71 to 4.09)	0.91 (0.77 to 1.09)
**Use of IUD or implant to prevent pregnancy at last sexual intercourse (n = 5,171)**	1.7 (0.8 to 3.6)	1.9 (0.9 to 4.1)	0.22 (–1.73 to 2.18)	1.13 (0.39 to 3.29)	4.7 (3.4 to 6.5)**	11.0 (7.3 to 16.2)^††^	6.29 (1.62 to 10.96)^§§^	2.34 (1.40 to 3.91)^¶¶^	7.4 (4.0 to 13.3)***	10.5 (6.2 to 17.4)^§§§^	3.12 (–3.97 to 10.21)	1.42 (0.64 to 3.15)
**Use of shot, patch, or birth control ring to prevent pregnancy at last sexual intercourse (n = 4,796)**	2.4 (1.1 to 5.3)	2.7 (1.4 to 5.3)	0.34 (–2.33 to 3.00)	1.14 (0.40 to 3.29)	3.3 (2.1 to 5.2)	3.7 (2.7 to 5.1)	0.45 (–1.49 to 2.39)	1.14 (0.65 to 2.00)	5.0 (2.9 to 8.3)	3.8 (1.8 to 7.9)	–1.18 (–5.01 to 2.65)	0.76 (0.31 to 1.90)
**Use of birth control pills to prevent pregnancy at last sexual intercourse (n = 5,171)**	14.8 (10.8 to 20.0)	11.7 (8.6 to 15.8)	–3.13 (–8.95 to 2.69)	0.79 (0.51 to 1.22)	24.2 (20.5 to 28.3)**	23.1 (20.4 to 26.0)^††^	–1.09 (–5.92 to 3.74)	0.96 (0.78 to 1.17)	27.5 (21.7 to 34.2)***	25.3 (19.6 to 32.1)^§§§^	–2.18 (–11.05 to 6.70)	0.92 (0.66 to 1.29)
**Use of no contraceptive method to prevent pregnancy at last sexual intercourse (n = 5,171)**	12.4 (8.6 to 17.6)	20.5 (16.9 to 24.6)	8.01 (2.11 to 13.92)^§^	1.64 (1.10 to 2.47)^¶^	10.5 (8.4 to 13.0)	11.1 (9.3 to 13.2)^††^	0.63 (–2.40 to 3.65)	1.06 (0.80 to 1.41)	9.7 (6.6 to 14.1)	13.1 (8.6 to 19.4)^§§§^	3.35 (–3.17 to 9.87)	1.34 (0.77 to 2.35)

**Abbreviations:** IUD = intrauterine device; PD= prevalence difference; PR = prevalence ratio; STD = sexually transmitted disease.

* 2019: N = 13,677 respondents; 2021: N = 17,232 respondents. Because the state and local questionnaires differ by jurisdiction, students in these schools were not asked all national YRBS questions. Therefore, the total number (N) of students answering each question varied. Percentages in each category are calculated on the known data. Unweighted counts indicating denominators. For HIV testing (lifetime), all students were included in the sample. For any condom use at last sexual intercourse and STD testing (past 12 months), sample was restricted to currently sexually active (i.e., having had sexual intercourse with at least one person during the 3 months before the survey). For use of IUD or implant, shot, patch, or birth control ring, birth control pills, and no contraceptive method to prevent pregnancy, sample was restricted to currently sexually active and those reporting opposite sex or both sex sexual contacts (lifetime).

† PD and PR compare 2019 versus 2021. 95% CIs that did not cross zero (for PD) or 1.0 (for PR) were considered statistically significant (p<0.05).

^§^ PD comparing 2019 versus 2021 among students aged ≤15 years indicated a significant difference (p<0.05).

^¶^ PR comparing 2019 versus 2021 among students aged ≤15 years indicated a significant difference (p<0.05).

** Students aged 16–17 years significantly differed from students aged ≤15 years in 2019, based on *t*-test with Taylor series linearization (p<0.05).

^††^ Students aged 16–17 years significantly differed from students aged ≤15 years in 2021, based on *t*-test with Taylor series linearization (p<0.05).

^§§^ PD comparing 2019 versus 2021 among students aged 16–17 years indicated a significant difference (p<0.05).

^¶¶^ PR comparing 2019 versus 2021 among students aged 16–17 years indicated a significant difference (p<0.05).

*** Students aged ≥18 years significantly differed from students aged ≤15 years in 2019, based on *t*-test with Taylor series linearization (p<0.05).

^†††^ Students aged ≥18 years significantly differed from students aged 16–17 years in 2019, based on *t*-test with Taylor series linearization (p<0.05).

^§§§^ Students aged ≥18 years significantly differed from students aged ≤15 years in 2021, based on *t*-test with Taylor series linearization (p<0.05).

^¶¶¶^ PD comparing 2019 versus 2021 among students aged ≥18 indicated a significant difference (p<0.05).

**** PR comparing 2019 versus 2021 among students aged ≥18 indicated a significant difference (p<0.05).

^††††^ Condom use at last sexual intercourse was measured by a separate item from the item for primary contraceptive method used for preventing pregnancy.

In 2021, students aged 16–17 years, compared with students aged ≤15 years, were more likely to report HIV testing (7.2% versus 3.5%), STD testing (18.2% versus 8.4%), IUD or implant use (11.0% versus 1.9%), and use of birth control pills (23.1% versus 11.7%), and less likely to report no contraceptive method use (11.1% versus 20.5%). In 2021, compared with students aged ≤15 years, students aged ≥18 years were more likely to report HIV testing (10.3% versus 3.5%), IUD or implant use (10.5% versus 1.9%), and use of birth control pills (25.3% versus 11.7%), and less likely to report condom use at last sexual intercourse (46.4% versus 55.1%) and no contraceptive method use (13.1% versus 20.5%).

Lifetime HIV testing decreased significantly for Black (PD = –6.47), Hispanic (PD = −3.15), and White students (PD = −3.17) (Table 4). Among sexually active White students, STD testing decreased significantly by 6.86 percentage points and did not significantly change for Black and Hispanic students. IUD or implant use increased significantly by 5.75 percentage points for Hispanic students. Among White students, no contraceptive method use increased significantly by 2.71 percentage points and did not significantly change for Black and Hispanic students. In 2021, compared with White students, Hispanic and Black students were less likely to use birth control pills (24.9%, 15.7%, 11.0%, respectively) and more likely to report no contraceptive method use (9.5%, 19.0%, 21.4%, respectively). In 2021, compared with Hispanic students, White students were less likely to report shot, patch, or birth control ring use (3.9% versus 2.2%) and more likely to report HIV testing (4.8% versus 6.6%) and STD testing (12.5% versus 19.5%).

**TABLE 4:** Changes in prevalence of sexual and reproductive health services and sexual behaviors among high school students, by race and ethnicity — Youth Risk Behavior Survey, United States, 2019–2021

Sexual and reproductive health services and sexual behavior (n)^†^	Black or African American*				Hispanic or Latino*				White*			
	2019 % (95% CI)	2021 % (95% CI)	PD % (95% CI)^§^	PR % (95% CI)^§^	2019 % (95% CI)	2021 % (95% CI)	PD % (95% CI)^§^	PR % (95% CI)^§^	2019 % (95% CI)	2021 % (95% CI)	PD % (95% CI)^§^	PR % (95% CI)^§^
**HIV testing, lifetime (n = 25,033)**	14.0 (11.3 to 17.1)	7.5 (5.7 to 9.7)	–6.47 (–9.98 to –2.95)^¶^	0.54 (0.38 to 0.75)**	9.7 (7.3 to 12.9)^††^	6.6 (5.5 to 7.8)^¶¶^	–3.15 (–6.16 to –0.13)***	0.68 (0.48 to 0.95)^†††^	8.0 (7.1 to 8.9)^§§§^	4.8 (3.8 to 6.0)	–3.17 (–4.57 to –1.78)^¶¶¶^	0.60 (0.47 to 0.77)****
**STD testing, past 12 months (n = 4,559)**	23.8 (16.8 to 32.5)	18.6 (11.6 to 28.6)	–5.12 (–16.67 to 6.44)	0.78 (0.45 to 1.38)	19.7 (13.8 to 27.2)	19.5 (15.5 to 24.2)^¶¶^	–0.18 (–8.15 to 7.78)	0.99 (0.66 to 1.49)	19.3 (16.2 to 22.9)	12.5 (10.1 to 15.3)	–6.86 (–11.06 to –2.66)^¶¶¶^	0.65 (0.49 to 0.84)****
**Condom use at last sexual intercourse (n = 6,455)** ^††††^	48.2 (43.3 to 53.2)	48.8 (41.8 to 55.8)	0.59 (–8.03 to 9.22)	1.01 (0.85 to 1.21)	56.2 (52.1 to 60.2)^††^	49.7 (43.8 to 55.6)	–6.50 (–13.72 to 0.72)	0.88 (0.77 to 1.02)	55.8 (53.0 to 58.6)^§§§^	54.6 (51.4 to 57.6)	–1.25 (–5.44 to 2.94)	0.98 (0.91 to 1.05)
**Use of IUD or implant to prevent pregnancy at last sexual intercourse (n = 5,171)**	2.0 (1.0 to 3.9)	6.2 (3.2 to 11.6)	4.20 (–0.02 to 8.41)	3.11 (1.22 to 7.93)**	1.6 (0.7 to 3.4)^§§^	7.3 (4.4 to 11.9)	5.75 (1.90 to 9.61)***	4.69 (1.85 to 11.89)^†††^	6.7 (5.0 to 8.9)^§§§^	10.0 (6.5 to 15.1)	3.31 (–1.32 to 7.94)	1.49 (0.90 to 2.48)
**Use of shot, patch, or birth control ring to prevent pregnancy at last sexual intercourse (n = 4,796)**	5.5 (3.0 to 10.0)	3.7 (2.4 to 5.8)	–1.81 (–5.54 to 1.92)	0.67 (0.32 to 1.43)	1.4 (0.6 to 3.2)^††,§§^	2.2 (1.5 to 3.3)^¶¶^	0.79 (–0.67 to 2.26)	1.56 (0.62 to 3.94)	4.3 (2.7 to 6.9)	3.9 (2.8 to 5.5)	–0.38 (–2.82 to 2.05)	0.91 (0.51 to 1.63)
**Use of birth control pills to prevent pregnancy at last sexual intercourse (n = 5,171)**	12.1 (8.8 to 16.4)	11.0 (7.5 to 15.9)	–1.03 (–6.66 to 4.59)	0.91 (0.56 to1.49)	15.5 (11.6 to 20.4)^§§^	15.7 (13.0 to 18.8)^¶¶^	0.18 (–5.08 to 5.43)	1.01 (0.72 to 1.42)	29.7 (25.8 to 33.9)^§§§^	24.9 (21.4 to 28.7)^§§§§^	–4.79 (–10.29 to 0.70)	0.84 (0.69 to 1.03)
**Use of no contraceptive method to prevent pregnancy at last sexual intercourse (n = 5,171)**	23.2 (19.3 to 27.6)	21.4 (15.2 to 29.2)	–1.76 (–9.92 to 6.40)	0.92 (0.64 to 1.34)	12.8 (9.1 to 17.7)^††,§§^	19.0 (14.6 to 24.4)^¶¶^	6.19 (–0.29 to 12.67)	1.48 (0.98 to 2.26)	6.8 (5.3 to 8.5)^§§§^	9.5 (7.7 to 11.5)^§§§§^	2.71 (0.24 to 5.17)^¶¶¶^	1.40 (1.03 to 1.91)****

**Abbreviations:** IUD = intrauterine device; PD= prevalence difference; PR = prevalence ratio; STD = sexually transmitted disease.

* Persons of Hispanic or Latino (Hispanic) origin might be of any race but are categorized as Hispanic; all racial groups are non-Hispanic.

^†^ 2019: N = 13,677 respondents; 2021: N = 17,232 respondents. Because the state and local questionnaires differ by jurisdiction, students in these schools were not asked all national YRBS questions. Therefore, the total number (N) of students answering each question varied. Percentages in each category are calculated on the known data. Unweighted counts indicating denominators. For HIV testing (lifetime), all students were included in the sample. For any condom use at last sexual intercourse and STD testing (past 12 months), sample was restricted to currently sexually active (i.e., having had sexual intercourse with at least one person during the 3 months before the survey). For use of IUD or implant, shot, patch, or birth control ring, birth control pills, and no contraceptive method to prevent pregnancy, sample was restricted to currently sexually active and those reporting opposite sex or both sex sexual contacts (lifetime).

^§^ PD and PR comparing 2019 versus 2021. 95% CIs that did not cross zero (for PD) or 1.0 (for PR) were considered statistically significant (p<0.05).

^¶^ PD comparing 2019 versus 2021 among Black or African American (Black) students indicated a significant difference (p<0.05).

** PR comparing 2019 versus 2021 among Black students indicated a significant difference (p<0.05).

^††^ Hispanic students significantly differed from Black students in 2019, based on *t*-test with Taylor series linearization (p<0.05).

^§§^ Hispanic students significantly differed from White students in 2019, based on *t*-test with Taylor series linearization (p<0.05).

^¶¶^ Hispanic students significantly differed from White students in 2021, based on *t*-test with Taylor series linearization (p<0.05).

*** PD comparing 2019 versus 2021 among Hispanic students indicated a significant difference (p<0.05).

^†††^ PR comparing 2019 versus 2021 among Hispanic students indicated a significant difference (p<0.05).

^§§§^ White students significantly differed from Black students in 2019 based on *t*-test with Taylor series linearization (p<0.05).

^¶¶¶^ PD comparing 2019 versus 2021 among White students indicated a significant difference (p<0.05).

**** PR comparing 2019 versus 2021 among White students indicated a significant difference (p<0.05).

^††††^ Condom use at last sexual intercourse was measured by a separate item from the item for primary contraceptive method used for preventing pregnancy.

^§§§§^ White students significantly differed from Black students in 2021, based on *t*-test with Taylor series linearization (p<0.05).

HIV testing significantly decreased for students reporting opposite sex only (PD = –2.96), same sex only (PD = −8.49), and both sexes sexual contacts (PD = −5.97) (Table 5). STD testing decreased significantly by 4.57 and 18.00 percentage points among students reporting opposite sex only and same sex only sexual contacts, respectively. IUD or implant use significantly increased by 8.14 percentage points among students reporting sexual contacts of both sexes and did not significantly change for students reporting opposite sex only contacts. In 2021, compared with students reporting opposite sex sexual contacts, students reporting both sexes sexual contacts were more likely to report HIV testing (9.7% versus 14.7%), STD testing (14.4% versus 23.4%), and no contraceptive method use (11.8% versus 23.7%), and less likely to report condom use at last sexual intercourse (55.5% versus 40.3%). In 2021, compared with students reporting same sex only contacts, students reporting both sexes sexual contacts were more likely to report STD testing (9.7% versus 23.4%) and condom use (18.3% versus 40.3%). In 2021, students reporting same sex only sexual contacts were less likely to report condom use than students reporting opposite sex only sexual contacts (18.3% versus 55.5%).

**TABLE 5:** Changes in prevalence of sexual and reproductive health services and sexual behaviors among high school students, by sex of sexual contacts — Youth Risk Behavior Survey, United States, 2019–2021

Sexual and reproductive health services and sexual behavior (n)*	Opposite sex only				Same sex only				Both sexes			
	2019 % (95% CI)	2021 % (95% CI)	PD % (95% CI)^†^	PR % (95% CI)^†^	2019 % (95% CI)	2021 % (95% CI)	PD % (95% CI)^†^	PR % (95% CI)^†^	2019 % (95% CI)	2021 % (95% CI)	PD % (95% CI)^†^	PR % (95% CI)^†^
**HIV testing, lifetime (n = 25,033)**	12.6 (11.1 to 14.3)	9.7 (8.3 to 11.2)	–2.96 (–5.14 to –0.78)^§^	0.77 (0.63 to 0.93)^¶^	18.5 (12.5 to 26.7)	10.1 (7.1 to 14.2)	–8.49 (–16.40 to –0.57)**	0.54 (0.32 to 0.91)^††^	20.7 (16.9 to 25.2)^§§^	14.7 (11.5 to 18.7)^¶¶^	-5.97 (–11.47 to 0.46)***	0.71 (0.52 to 0.98)^†††^
**STD testing, past 12 months (n = 4,559)**	19.0 (16.3 to 21.9)	14.4 (12.1 to 17.0)	–4.57 (–8.27 to –0.88)^§^	0.76 (0.61 to 0.95)^¶^	27.7 (15.6 to 44.1)	9.7 (4.9 to 18.3)	–18.00 (–33.87 to –2.14)**	0.35 (0.15 to 0.82)^††^	29.7 (23.1 to 37.3)^§§^	23.4 (17.2 to 30.9)^¶¶,§§§^	–6.37 (–16.26 to 3.52)	0.79 (0.54 to 1.15)
**Condom use at last sexual intercourse (n = 6,455)^¶¶¶^**	56.3 (53.7 to 58.8)	55.5 (52.8 to 58.3)	–0.73 (–4.48 to 3.02)	0.99 (0.92 to 1.06)	29.0 (17.7 to 43.6)****	18.3 (8.0 to 36.6)^††††^	–10.66 (–29.98 to 8.65)	0.63 (0.26 to 1.55)	45.3 (37.1 to 53.7)^§§^	40.3 (32.0 to 49.1)^¶¶,§§§^	–4.99 (–17.02 to 7.03)	0.89 (0.67 to 1.18)
**Use of IUD or implant to prevent pregnancy at last sexual intercourse (n = 5,171)**	4.8 (3.3 to 7.0)	8.3 (5.4 to 12.5)	3.50 (–0.44 to 7.45)	1.73 (0.98 to 3.04)	—^§§§§^	—	—	—	4.8 (2.5 to 8.9)	12.9 (8.9 to 18.3)	8.14 (2.59 to 13.68)***	2.70 (1.31 to 5.56)^†††^
**Use of shot, patch, or birth control ring to prevent pregnancy at last sexual intercourse (n = 4,796)**	3.6 (2.5 to 5.1)	3.6 (2.8 to 4.6)	0.04 (–1.51 to 1.60)	1.01 (0.66 to 1.56)	—	—	—	—	2.7 (1.0 to 6.9)	3.0 (1.5 to 5.8)	0.24 (–3.04 to 3.52)	1.09 (0.34 to 3.49)
**Use of birth control pills to prevent pregnancy at last sexual intercourse (n = 5,171)**	23.5 (19.9 to 27.6)	21.4 (18.8 to 24.3)	–2.14 (–6.87 to 2.60)	0.91 (0.74 to 1.12)	—	—	—	—	21.0 (14.6 to 29.1)	16.7 (11.3 to 23.8)	–4.30 (–13.86 to 5.26)	0.79 (0.48 to 1.32)
**Use of no contraceptive method to prevent pregnancy at last sexual intercourse (n = 5,171)**	9.4 (7.7 to 11.5)	11.8 (10.3 to 13.6)	2.39 (–0.12 to 4.90)	1.25 (0.98 to 1.60)	—	—	—	—	22.4 (15.7 to 30.9)^§§^	23.7 (19.8 to 28.2)^¶¶^	1.36 (–7.31 to 10.02)	1.06 (0.72 to 1.55)

**Abbreviations:** IUD = intrauterine device; PD= prevalence difference; PR = prevalence ratio; STD = sexually transmitted disease.

* 2019: N = 13,677 respondents; 2021: N = 17,232 respondents. Because the state and local questionnaires differ by jurisdiction, students in these schools were not asked all national YRBS questions. Therefore, the total number (N) of students answering each question varied. Percentages in each category are calculated on the known data. Unweighted counts indicating denominators. For HIV testing (lifetime), all students were included in the sample. For any condom use at last sexual intercourse and STD testing (past 12 months), sample was restricted to currently sexually active (i.e., having had sexual intercourse with at least one person during the 3 months before the survey). For use of IUD or implant, shot, patch, or birth control ring, birth control pills, and no contraceptive method to prevent pregnancy, sample was restricted to currently sexually active and those reporting opposite sex or both sex sexual contacts (lifetime).

^†^ PD and PR comparing 2019 versus 2021. 95% CIs that did not cross the null value of 0 (for PD) or 1.0 (for PR) were considered statistically significant (p<0.05).

^§^ PD comparing 2019 versus 2021 among students having opposite sex only sexual contacts indicated a significant difference (p<0.05).

^¶^ PR comparing 2019 versus 2021 among students having opposite sex only sexual contacts indicated a significant difference (p<0.05).

** PD comparing 2019 versus 2021 among students having same sex only sexual contacts indicated a significant difference (p<0.05).

^††^ PR comparing 2019 versus 2021 among students having same sex only sexual contacts indicated a significant difference (p<0.05).

^§§^ Students having both sex sexual contacts significantly different from students having opposite sex only sexual contacts in 2019, based on *t*-test with Taylor series linearization (p<0.05).

^¶¶^ Students having both sex sexual contacts significantly different from students having opposite sex only sexual contacts in 2021, based *t*-test with Taylor series linearization (p<0.05).

*** PD comparing 2019 versus 2021 among students having both sex sexual contacts indicated a significant difference (p<0.05).

^†††^ PR comparing 2019 versus 2021 among students having both sex sexual contacts indicated a significant difference (p<0.05).

^§§§^ Students having both sex sexual contacts significantly different from students having same sex only sexual contacts in 2021, based on *t*-test with Taylor series linearization (p<0.05).

^¶¶¶^ Condom use at last sexual intercourse was measured by a separate item from the primary method used for preventing pregnancy item.

**** Students having same sex only sexual contacts significantly different from students having opposite sex only sexual contacts in 2019, based on *t*-test with Taylor series linearization (p<0.05).

^††††^ Students having same sex only sexual contacts significantly different from students having opposite sex only sexual contacts in 2021, based on *t*-test with Taylor series linearization (p<0.05).

^§§§§^ Prevalence estimates with a denominator <30 were considered statistically unreliable and therefore were suppressed.

## Discussion

This study provides the first nationally representative estimates of sexual behaviors and receipt of SRH services before and during the COVID-19 pandemic among U.S. high school students. Since the pandemic, considerable decreases in HIV and STD testing were identified, and the magnitude and presence of the decline in receipt of SRH services primarily accessible through health care providers varied by demographic characteristics, including sex, age, race and ethnicity, and sex of sexual contacts.

Declines in overall HIV testing among all adolescents and STD testing among those sexually active mirror similar reductions in STD/HIV testing, particularly among adolescent males who have sex with men in the United States during the pandemic period (*10*). Changes in sexual activity patterns and medical office closures or limited-service offerings might have affected testing during the pandemic (*10*). Overall, fewer than 10% of high school students reported HIV testing, and differences by age, race and ethnicity, and sex of sexual contacts illustrate gaps in meeting recommendations for universal and routine HIV screening for all youths aged ≥13 years at least once (https://www.cdc.gov/hiv/guidelines/testing.html). Students who reported same sex only and both sexes sexual contacts had the greatest decreases in HIV testing. This finding is concerning because of disproportionate rates of HIV infection among adolescent sexual minority males (*11*). Addressing structural barriers to HIV services for adolescents (e.g., access to culturally responsive and inclusive testing services) remains a priority.

From 2019 to 2021, sexually active younger students (aged ≤15 years) and male students were less likely to have received STD testing than older and female students; however, female and older students had larger declines in STD testing than these groups. Higher prevalence of STD screening among sexually active female students compared with male students might be explained by greater rates of chlamydia and gonorrhea among females and aligns with recommendations for annual chlamydia and gonorrhea screening for sexually active women aged ≥25 years (https://www.cdc.gov/std/treatment-guidelines/default.htm). White students reported the largest decrease in STD testing (6%) and were less likely to have received STD testing than Hispanic students in 2021. Similar patterns are observed in sex-stratified data from the National Survey of Family Growth (NSFG) (2013–2019), wherein Black and Hispanic males received STD tests at larger proportions than White males (*12*). Students with same sex only sexual contacts experienced the largest decrease in STD testing, mirroring other estimates of declines in STI/HIV testing among adolescent males who have sex with males and other sexual minority groups (*10*).

Improving access to STD testing is important because of persistent and disproportionate increases in rates of infection (https://www.cdc.gov/std/statistics/2021/default.htm) and suboptimal adherence to current CDC recommendation for a certain level of STD screening among adolescents. Delays or elimination of clinical care services, shortages, supply issues regarding testing materials (e.g., self-testing kits), and hesitancy in seeking health services have affected testing during the pandemic (*7*). Continuing to identify STD testing needs and providing routine screening and testing throughout adolescence, while considering confidentiality concerns (*13*), annual screening guidelines, and pandemic disruptions, is needed. The updated CDC STD Strategic Plan 2022–2026 (https://www.cdc.gov/std/dstdp/dstdp-strategic-plan-2022-2026.htm) and National HIV/AIDS Strategy (https://www.whitehouse.gov/wp-content/uploads/2021/11/National-HIV-AIDS-Strategy.pdf) provide guidance for addressing disparities among persons with varying racial and ethnicity and sexual identities, including strategies for screening, testing, and treatment in a variety of clinical and community settings.

Among the contraceptive methods used to prevent pregnancy at last sexual intercourse examined in this study, certain changes over time were identified among specific subgroups. The use of no contraceptive method significantly increased from 10.7% to 13.4% and was greatest among students aged ≤15 years and White students. Younger students reporting higher use of no contraceptive method mirrors prepandemic trends (2015–2019) among sexually active adolescents, illustrating persistent patterns of higher contraceptive method nonuse among those aged 15–17 versus 18–19 years (*2*). However, there was a significant increase (4.8% to 8.9%) in IUD or implant use. The increase in prevalence of these long-acting reversible contraceptive (LARC) methods is noteworthy and parallels other nationwide prepandemic trends in increased use (*2*). Older adolescents were more likely to report LARC use, which aligns with prepandemic findings from the Title X National Family Planning Program (*14*). Improving awareness and counseling on all available contraceptives, including LARC methods, as sexually active adolescents age remains important (*13*). Hispanic and Black students reported increases in IUD or implant use from 2019 to 2021, illustrating progress toward addressing racial and ethnic disparities and improving use of highly effective methods of contraception in 2021. As access to SRH services improves as part of pandemic recovery, return of fully available clinic and school-based services; use of same-day initiation of LARC methods and counseling on the importance of STD/HIV testing; and contraceptive method choice, including using both condoms and hormonal contraceptive methods; might help reduce disparities (*4*). In addition, because of high prevalence of condom use for pregnancy prevention among sexually active students (*2*), future research examining trends and differences in use of condoms as the primary contraceptive method used at last sexual intercourse are warranted.

## Limitations

General limitations for YRBS are available in the overview report of this supplement (*9*). The findings in this report are subject to at least five additional limitations. First, limited sample size prevented stratifying by multiple characteristics concurrently (e.g., by sex and sex of sexual contacts) and examining race and ethnicity subgroups with smaller samples (e.g., Asians). Second, male students might not be aware of their female partner’s contraceptive use at last sexual intercourse (*15*). Third, separating condom use for pregnancy prevention versus STD/HIV prevention is not feasible. Although YRBS assesses condom use as a primary method for pregnancy prevention (not included), condom use for STD/HIV prevention is not explicitly measured. Fourth, differences in question wording (Table 1) might have affected comparability in questions and responses between 2019 and 2021; however, the study team used analytic strategies to address question comparability where possible (e.g., limited the analytic sample to exclude students with only same sex sexual contacts). Finally, in 2021, the response option for use of birth control pills explicitly said to exclude emergency contraception; however, in 2019, the response option did not. Students might have differentially classified emergency contraception in 2019 versus 2021. Data from the NSFG indicate steady increases in emergency contraception use among sexually active adolescents in the United States between 2008 and 2015 (14% and 21%, respectively) (*16*), highlighting an important contraceptive method to monitor through ongoing surveillance.

## Future Directions

The findings in this report highlight opportunities to promote adolescent sexual health by addressing pandemic-driven disruptions and historically declining trends in adolescent protective sexual behaviors (e.g., condom use) (*2*,*3*). Continuing to provide adolescent friendly SRH services delivered by culturally competent and trained providers is critical. Before and during the pandemic, innovative models of telehealth proved viable for ensuring the delivery of essential health services, including SRH services (*17*). For example, health care providers report using telehealth for contraception initiation or continuation and STD testing services during the pandemic (*18*), possibly affecting changes in select contraceptive methods used to prevent pregnancy among the sexually active high school students in this study. Continued work to address documented challenges in telehealth (e.g., confidentiality) for SRH service delivery (*13*,*18*), including maintaining availability of in-person services, remains important.

Schools are positioned to help support adolescent SRH by offering onsite or community-based preventive health services and comprehensive education (https://www.cdc.gov/healthyyouth/whatworks/what-works-overview.htm). Studies indicate increases in STD/HIV testing and hormonal contraceptive use among adolescents who attend schools with established health service referral systems (*19*). Tools and resources to help develop and implement referral systems that link adolescents to school- or community-based SRH services are available at https://www.ncsddc.org/resource/developing-a-referral-system-for-sexual-health-services-2/. One study also illustrates positive effects of STD/HIV and pregnancy prevention education on adolescent sexual risk and protective behaviors (*20*). Because of the study’s findings regarding high contraceptive method nonuse among students aged ≤15 years, explicit attention to building SRH knowledge and skills for using preventative clinical services among this cohort of adolescents is needed. CDC’s Health Education Curriculum Analysis Tool (HECAT) (https://www.cdc.gov/healthyyouth/hecat/pdf/2021/hecat_module_sh.pdf) can be used to address concerning adolescent sexual behaviors observed during the pandemic by guiding school-based delivery of comprehensive, developmentally appropriate, inclusive, and culturally responsive education.

Finally, youth- and community-driven initiatives that work to dismantle existing or new barriers to accessing and using SRH services are important. Implementation research suggests youths should be engaged as decision-makers in SRH program design and implementation (*21*). Working directly with adolescents and community members who experience disparate access to SRH services can help ensure needs are being met through culturally responsive and inclusive care.

## Conclusion

Access to quality, affordable, and confidential SRH services remains critical for reducing STD/HIV and unintended pregnancy among adolescents. Social and economic changes during the COVID-19 pandemic, including social distancing, stay-at-home orders, and health care facility and school closures, might have affected the sexual behaviors and use of a range of SRH services and resources for adolescents across the United States. Decreases in STD/HIV testing and increases in nonuse of contraception signal the continuing need for comprehensive, inclusive, and culturally responsive education and health services. Findings can be bolstered by future work on the long-term effects of COVID-19 on adolescent’s sexual behaviors, romantic relationships, and sexual health care.

## Footnotes

* See e.g., 45 C.F.R. part 46.102(l)(2), 21 C.F.R. part 56; 42 U.S.C. §241(d); 5 U.S.C. §552a; 44 U.S.C. §3501 et seq.
